# Breach and restoration of retinal immune privilege: barrier failure, innate dysregulation, and adaptive autoimmunity

**DOI:** 10.3389/fimmu.2025.1703382

**Published:** 2025-11-27

**Authors:** Qiwei Fan, Zhijie Li

**Affiliations:** 1 Department of Ophthalmology, Zhongshan Torch Development Zone People’s Hospital, Zhongshan, Guangdong, China; 2 Department of Ophthalmology, The First Affiliated Hospital of Jinan University, Guangzhou, China; 3 International Ocular Surface Research Center, Institute of Ophthalmology, and Key Laboratory for Regenerative Medicine, Jinan University, Guangzhou, China

**Keywords:** immune privilege, blood retinal barrier, microglia, complement, inflammasome, Th17/T cells, age-related macular degeneration, diabetic retinopathy

## Abstract

The retina preserves vision by tightly regulating inflammation (“immune privilege”) via blood–retinal barriers, neuroglial checkpoints, and tolerogenic cues. This actively maintained—and potentially restorable—state is breached in major retinal diseases through three recurrent archetypes. We synthesize 2015–2025 advances into a framework of barrier failure, innate dysregulation, and adaptive autoimmunity. Across age-related macular degeneration (AMD), diabetic retinopathy (DR), retinitis pigmentosa (RP), and non-infectious uveitis (NIU): AMD exhibits complement–microglia para-inflammation with later outer-barrier compromise; DR exemplifies inner-BRB failure with inflammatory amplification; RP begins with degeneration-triggered innate activation and progresses to combined innate–adaptive injury; NIU represents T-cell-driven breach of the blood–retinal barrier. Interventional human evidence supports immunity as a therapeutic target: complement inhibition slows geographic atrophy; anti-VEGF reduces leak; intravitreal corticosteroids suppress inflammatory edema; and anti-TNF/IL-6R improve refractory NIU. Emerging strategies aim at privilege restoration—reinforcing myeloid checkpoints, tempering inflammasomes, and exploring tolerance-oriented approaches to re-educate adaptive immunity. Evidence from preclinical and early translational studies indicates that ocular tissues can imprint regulatory/anergic programs on pathogenic T cells, supporting mechanism-aligned, patient-tailored immunotherapy as a testable route to restore regulation, mitigate inflammation, and slow degeneration.

## Highlights

- Retinal immune privilege is an active, dynamic state maintained by barriers, neuroglia, and inhibitory ligand–receptor checkpoints.
- Distinct breach archetypes—barrier failure, innate dysregulation, adaptive autoimmunity—drive AMD, DR, RP, and non-infectious uveitis, respectively.
- Complement–microglia para-inflammation is a core pathogenic axis in AMD and late-stage RP.
- Inner BRB breakdown in DR and T cell–driven barrier breach in uveitis illustrates divergent immune-mediated injury routes.
- Mechanism-aligned interventions span barrier stabilizers, complement/inflammasome inhibitors, microglial checkpoint reinforcement, and antigen-specific tolerance strategies aimed at restoring immune privilege.

## 1 Introduction

The retina is an immune-privileged neural tissue in which the blood–retinal barriers (BRBs) limit systemic immune traffic while local immunoregulatory programs actively suppress inflammatory activation to preserve vision (1, 2). In homeostasis, two BRBs—the tight-junction–sealed inner BRB (endothelium–pericyte–glia unit) and the outer BRB (RPE tight junctions)—restrict parenchymal access of plasma proteins and leukocytes (1, 2). The immune-quiescent milieu is reinforced by soluble mediators (notably TGF-β2 and α-MSH in ocular fluids) and membrane checkpoints (RPE-expressed PD-L1 and FasL) that dampen or delete incoming effector lymphocytes (3–7). Neuron–glia “braking” signals, including CD200–CD200R and CX3CL1–CX3CR1, maintain microglia in a surveillant, non-injurious state and calibrate innate responses to danger cues (8–13).

Immune privilege, however, is not absolute: it can be breached through three non-exclusive routes—(i) barrier failure with vascular leakage and immune extravasation, (ii) intrinsic dysregulation of resident microglia together with complement/inflammasome axes, and (iii) adaptive autoimmunity when retinal antigens are presented in immunogenic contexts (7, 14–17). This review applies that mechanistic “breach-archetype” framework to four major retinal disorders—age-related macular degeneration (AMD), diabetic retinopathy (DR), inherited retinal degeneration (exemplified by retinitis pigmentosa, RP), and non-infectious uveitis (NIU)—which span a spectrum from para-inflammatory innate activation to overt T cell autoimmunity (16, 18–20). In AMD, aging stress at the RPE–Bruch’s interface and complement dysregulation drive chronic, compartmentalized innate activation (microglia/macrophages and complement on drusen), with barrier compromise emerging mainly in neovascular stages (16, 18, 21). In DR, chronic hyperglycemia precipitates inner BRB failure (pericyte dropout, endothelial junction loss) and leukostasis, leading to neurovascular inflammation and edema (22–25). In RP, primary genetic photoreceptor death initiates microglial/complement-tagged clearance that can progress to outer-BRB loosening and peripheral CCR2^+^ monocyte/ T cell involvement, amplifying degeneration (26, 27). In autoimmune uveitis, peripherally primed Th17/Th1 cells infiltrate and actively breach the BRB, recruiting myeloid effectors to cause fulminant tissue injury (28–32).

Convergent insights from human studies, animal models, and systems immunology enable a unified synthesis of the cellular and molecular circuits that maintain privilege in health and drive pathology when compromised. Here we (i) map mechanisms to breach archetypes and dominant disease drivers, (ii) highlight seminal advances, and (iii) discuss emerging strategies to preserve or restore immune regulation, including barrier-stabilizing approaches, innate “noise” dampening, and antigen-focused tolerance tailored to disease context. We propose that privilege restoration—resealing BRBs, lowering innate set-points, and re-imposing adaptive tolerance—offers a coherent, mechanism-driven path to preventing vision loss across diverse retinal diseases.

## 2 Search strategy & scope

We searched PubMed, Web of Science Core Collection, and ClinicalTrials.gov from Jan 1, 2015 to Aug 15, 2025. Search strings combined retina-specific terms (*retina/retinal; blood–retinal barrier/BRB*), immune-mechanistic terms (*immune privilege; microglia/macrophage; complement/C3/CFH; inflammasome/NLRP3; tolerance/ACAID (associated immune deviation); Treg/Th17*), disease terms (*AMD; geographic atrophy; nAMD; diabetic retinopathy/diabetic macular edema; retinitis pigmentosa/inherited retinal degeneration; non-infectious uveitis*), and named interventions (*pegcetacoplan; avacincaptad; faricimab; adalimumab; tocilizumab; minocycline; CX3CL1; CD200R; polysialic acid*).

Inclusion criteria: interventional human, observational human, or preclinical studies that elucidate retinal immune privilege or its breach, or evaluate mechanism-aligned therapies. Exclusion criteria: case reports without mechanistic content, editorials/opinions without primary data, conference abstracts without full text. Backward/forward citation tracking was performed from key studies and reviews. Although our formal window is 2015–2025, we explicitly retain pre 2015 foundational work (e.g., ACAID and aqueous humor factors (3–5, 21, 33–38); FasL mediated privilege (6, 39); CD200–CD200R and CX3CL1–CX3CR1 checkpoints (8–13)) to anchor concepts and contextualize recent advances, with evidence labeling unchanged. Screening: two reviewers independently screened titles/abstracts and full texts; disagreements were adjudicated by a third reviewer.

Evidence is consistently labeled as Interventional human, Observational human, or Preclinical across the text and **Table 1**; causal wording is reserved for interventional human data, whereas observational/preclinical findings are treated as associative or hypothesis-generating. At the end of each section, we summarize in italics the nature of the evidence presented (e.g., interventional human, observational human, preclinical).

**Table 1:** Comparative mapping of immune privilege breach archetypes, dominant mechanisms, and therapeutic targets across AMD, DR, RP, and non-infectious uveitis.

Disease	Dominant breach archetype(s) (0-3)	Principal immune mechanisms (selected examples)	Representative targets / agents (stage)	Evidence nature
AMD	Innate (3) > Barrier (1–2), Adaptive (0–1)	Complement activation on drusen/RPE(I); subretinal microglia/macrophage clustering (CX3CR1-dependent) (I); inflammasome signals at RPE; age-related sialylation decline affecting complement “self” codes (13, 18) (I)	C3 (pegcetacoplan, approved) and C5 (avacincaptad, approved) — GA (approved) (18)(I); Anti VEGF: aflibercept 8 mg (q8–16 wk), faricimab (up to q16 wk) — CNV (approved; barrier resealing) (71, 72, 111)(I); NLRP3 inhibitors (exploratory) (18)(I); glycocode restoration (polysialic acid, exploratory) (18) (I)	Interventional human (pegcetacoplan, anti-VEGF); Observational human (genetics, tissue); Preclinical (CX3CR1, glycocode, inflammasome)
DR	Barrier (3) + Innate (2), Adaptive (1)	Pericyte loss & endothelial TJ breakdown → inner BRB failure (22, 23)(B); leukostasis (neutrophils/monocytes) → capillary closure (73–75)(B); intraocular IL-6/IL-8/CCL2 ↑ with stage (76–79)(B); inflammasome/IL-1β drives capillary degeneration (75)(B)	Anti-VEGF for DME/NPDR–PDR (80)(B); Steroids (DEX/FA implants) (19)(B); IL-6R blockade (tocilizumab) for refractory DME/uveitis overlap (clinical/phase trials) (81)(B); NLRP3 inhibition (exploratory) (82)(B)	Interventional human (anti-VEGF, steroids); Observational human (IL-6 correlations); Preclinical (IL-1β/caspase-1, NLRP3)
RP (IRD)	Innate (2–3, early) → Barrier (1–2, late) ± Adaptive (1–2)	Degeneration-triggered microglial activation & migration before cell loss (46–49, 83)(I/A); decreased TGF-β1/PD-L1 and TJ proteins, loosening outer BRB (26)(I/A); CCR2^+^ monocyte and T cell infiltration (26, 27)(I/A); anti-retinal antibodies in subsets (84, 85, 93)(I/A)	Minocycline — reduces microglia/CCR2^+^ recruitment(I/A); photoreceptor protection in mouse; small open-label human trial reports functional benefit (exploratory) (27, 94)(I/A); Complement/NLRP3 modulation (exploratory) (18)(I/A); CD200R axis enhancement (preclinical, CNV models) (60)(I/A)	Preclinical (mechanistic mouse models); Interventional human (exploratory, open-label); Observational human (tissue/cytokine changes)
Non-infectious uveitis	Adaptive (3) + Barrier (2) + Innate (1–2)	Th17/Th1 infiltration; granzyme-mediated TJ cleavage(A); cytokine milieu (IL-17, IFN-γ, TNF-α) (28, 98)(A); a few thousand IRBP-specific T cells sufficient for full EAU (30, 31)(A); bone-marrow resident memory sustains relapse (99, 100)(A)	TNF-α inhibitor (adalimumab) — refractory uveitis (approved, VISUAL trials) (101)(A); IL-6R blockade (tocilizumab) — macular edema/inflammation (clinical/phase) (81)(A); IL-17 inhibition (mixed outcomes) (28)(A); Treg/Breg tolerance protocols (preclinical) (102, 103)(A)	Interventional human (anti-TNF, IL-6R blockade); Observational human (immune profiling); Preclinical (EAU, tolerance strategies)

The Dominant Breach Archetype scores are semi-quantitative heuristic values on a 0–3 scale reflecting the relative dominance of each archetype in the disease’s pathogenesis. The scores are defined as: 0 = minimal, 1 = minor, 2 = contributory, 3 = dominant).

Multiple badges denote cross axis relevance (grayscale fallback: superscripts B/I/A) These scores synthesize the weight of evidence rather than derive from a formal meta-analysis; their purpose is to orient the reader to the relative contribution of each breach archetype in a given disease, with disease and stage specific detail developed in Sections 5–8.

## 3 Retinal immune privilege in health

### 3.1 Resident immune and glial cells of the retina

Throughout this review, “microglia” refers to yolk-sac–derived, self-renewing parenchymal myeloid cells, whereas “macrophages” denotes monocyte-derived cells recruited from bone-marrow precursors and are recruited as CCR2^+^ intermediates when BRBs loosen or inflammatory cues rise; the terms are not interchangeable (21, 27, 40–46).

Composition and ontogeny. The healthy retina contains a small but indispensable network of resident immune cells—principally microglia within the neural retina and tissue macrophages in adjacent ocular structures (perivascular, choroidal, iris/ciliary body macrophages) (40). Retinal microglia derive from primitive yolk sac progenitors that colonize the tissue during embryogenesis (40), then self-renew locally and persist across the lifespan with minimal input from circulating monocytes (41–45).

Homeostatic surveying and housekeeping. In health, microglia adopt a highly ramified morphology and continuously scan the parenchyma with motile processes (46–49). They contribute to synaptic pruning, efferocytic clearance of apoptotic material, and secrete neurotrophic factors that sustain neurons (46–49). Microglia cluster within the outer and inner plexiform layers, an anatomical positioning that facilitates synaptic modulation and rapid response to injury (46–50).

Neuron–microglia inhibitory checkpoints. Retinal neurons and vascular endothelium constitutively express CD200, which ligates CD200R on microglia/macrophages to restrain activation, maintaining a surveillant but non-inflammatory state (8–10). Neurons also display membrane-anchored CX3CL1 (fractalkine), with CX3CR1 selectively expressed by microglia; CX3CL1–CX3CR1 signaling curbs microglial phagocytosis and cytokine release, while disruption exaggerates injury phenotypes (11–13, 51). Together these contact-dependent checkpoints are central to microglial quiescence and spatial tuning across retinal layers (8–13, 50, 51).

Macroglia as immune regulators. Müller glia span the full retinal thickness and astrocytes populate the nerve fiber and perivascular layers; both reinforce the inner BRB and shape immune tone (52). Müller cells secrete neurotrophic and anti-inflammatory mediators (e.g., PEDF, fibulin) and release TGF-β and related factors that suppress immune activation (52). By buffering extracellular glutamate/ATP and via direct cell–cell interactions, Müller glia and astrocytes help retain microglia in a restrained program (52). Importantly, TGF-β signaling is required cell-intrinsically in microglia; its loss precipitates maladaptive activation and degeneration, underscoring a glia–myeloid regulatory axis (53).

RPE as an immune gatekeeper. The retinal pigment epithelium (RPE) forms the outer BRB through tight junctions, regulating molecular and cellular traffic from the fenestrated choroid (3–5, 54). RPE constitutively secrete TGF-β2, α-MSH, and PEDF, establishing a localized anti-inflammatory milieu at the subretinal interface (3–5, 54). They also express PD-L1 and FasL, which can delete or energize infiltrating lymphocytes and promote tolerogenic fates in APCs/T cells (3–5, 54). Ocular vascular endothelium contributes by expressing FasL along vascular walls, adding an additional “do-not-attack” signal (6, 52, 55).

Microglia, perivascular/subretinal macrophages, Müller cells, astrocytes, and the RPE operate as an interlocked consortium: they maintain physical sequestration (inner/outer BRBs) and broadcast suppressive cues that bias local immunity toward tolerance (3–6, 52, 54, 55). This combination of anatomical separation and active regulation embodies ocular immune privilege, constraining inflammation to preserve neural function (6, 7, 14, 15, 55, 56).

Evidence: lineage tracing and fate-mapping studies in animal models, supported by limited human transcriptomic confirmation; in vivo imaging and experimental models, with associative support from human post-mortem analyses; murine knock-out/knock-in models, with growing human genetic associations; causality in human disease remains inferential; conditional knockout mouse models; translation to human pathophysiology is plausible but not yet confirmed; ex vivo RPE and endothelial cultures and in vivo models; direct demonstration in human disease contexts is still emerging. Consensus is supported by convergent human and animal evidence, though mechanistic causality remains best defined in experimental systems.

### 3.2 Mechanisms maintaining immune privilege

Several overlapping mechanisms collectively protect the retina from immune-mediated damage, acting through barriers, soluble cues, inhibitory checkpoints, and systemic tolerance.

#### 3.2.1 Blood–retinal barriers

The retina’s immune sequestration rests on two interlocking barriers: the inner BRB formed by non-fenestrated endothelium with tight junctions supported by pericytes and perivascular glia (neurovascular unit), and the outer BRB constituted by RPE tight junctions separating neural retina from fenestrated choroidal capillaries (1, 2). Functionally, these barriers exclude plasma proteins and leukocytes under physiological conditions, permitting only tightly regulated transport and surveillance (1, 2). Barrier competence is reinforced by glia–endothelium signaling (e.g., Wnt/β-catenin), underscoring that integrity is actively maintained rather than purely anatomical (57). Clinically and experimentally, the vulnerability of the inner BRB becomes evident when pericytes are lost—an early lesion in diabetes that precedes frank vasculopathy and leak—illustrating how pericyte support is integral to barrier function (22, 23).

#### 3.2.2 Immunosuppressive factors in ocular fluids

Ocular compartments (aqueous humor; retinal interstitium) are enriched for immunosuppressive mediators that restrain T cells and phagocytes and bias toward regulatory phenotypes, notably TGF-β2 and α-MSH detected in human and animal aqueous humor (3–5, 54). Mechanistically, TGF-β can suppress microglial activation *in vitro*, and loss of TGF-β signaling in retinal microglia precipitates degeneration and worsens choroidal neovascularization *in vivo* (53, 58). Beyond soluble cytokines, RPE-derived vesicles and membranes can induce macrophage apoptosis, providing a cell-extrinsic route to silence effectors at the retina–choroid interface (52). Collectively, these aqueous/retinal fluid factors generate a soluble “privilege bath” that lowers the activation set-point of resident and infiltrating immune cells (3–5, 52–54, 58).

#### 3.2.3 Inhibitory ligand–receptor checkpoints

**Figure 1:**
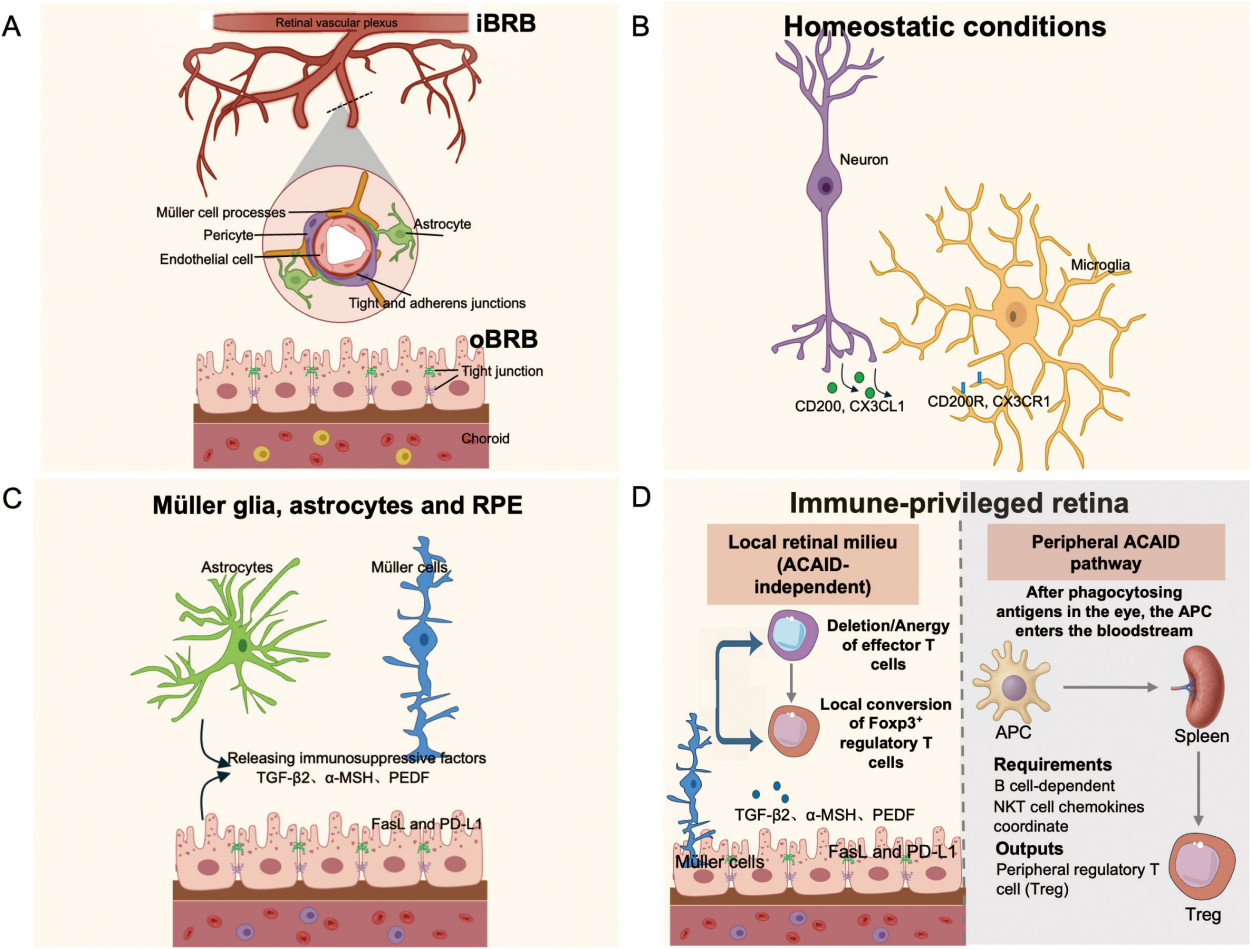
Immune privilege of the healthy retina. **(A)** The inner and outer blood–retinal barriers (BRBs) restrict entry of plasma proteins and leukocytes, maintaining immune sequestration. **(B)** Homeostatic microglia (green) adopt a ramified, surveillant morphology within the plexiform layers. Neuronal ligands such as CD200 and CX3CL1 signal to microglial receptors (CD200R, CX3CR1) to restrain activation; disruption of these pathways promotes degeneration, while augmentation can preserve cone function. **(C)** Macroglia, including Müller cells and astrocytes, support barrier function and modulate immune tone. The retinal pigment epithelium (RPE) secretes immunosuppressive mediators (e.g., TGF-β2, α-MSH) and expresses inhibitory ligands (FasL, PD-L1) that dampen or delete infiltrating effectors. **(D)** Left-Local retinal milieu: RPE/Müller/vascular cells express FasL/PD L1 (driving deletion/anergy) and release TGF β_2_, α MSH, PEDF (promoting local conversion to Foxp3^+^ Tregs), thereby reprogramming effector T cells independent of ACAID. Right-Peripheral ACAID pathway: ocular APCs migrate to the spleen (ACAID; B cell–dependent; NKT cell chemokines coordinate) to induce systemic regulatory responses. Together, peripheral ACAID and local on-site imprinting cooperate to sustain ocular immune privilege.

Neural and barrier-forming cells provide tonic “calm-down” signals to microglia and infiltrating leukocytes (**Figure 1**). The CD200–CD200R dyad restrains macrophage/microglial activation; retinal CD200 limits microglial activation and tissue damage in EAU models (8, 10). In parallel, the CX3CL1–CX3CR1 (fractalkine) axis maintains microglia in a surveillant state; genetic loss of CX3CR1 heightens phagocytosis and accelerates degeneration/para-inflammation, whereas augmenting soluble CX3CL1 (AAV-sCX3CL1 gene therapy) preserves cone function in degenerating retinas (11, 12, 51, 59). These checkpoints are therapeutically tractable: agonizing CD200R suppresses pro-angiogenic macrophage programs and reduces choroidal neovascularization *in vivo (*60, 61). In addition, Fas ligand (FasL) and PD-L1 expressed by ocular tissues can delete or inactivate infiltrating effectors, providing a membrane-bound safeguard (6, 39). Together these ligand–receptor brakes stabilize tissue homeostasis and prevent persistent microglial or T cell activation (6, 8, 10–13, 39, 51, 59–61).

#### 3.2.4 Immune deviation and tolerance

The eye engages both systemic and local mechanisms to restrain destructive adaptive responses to sequestered retinal antigens (**Figure 1**). In anterior chamber, associated immune deviation (ACAID), ocular antigen presenting cells (APCs) acquire antigen intraocularly and traffic to the spleen, where they drive a deviation toward regulation rather than effector immunity. An intact spleen and B cell compartment are required for full expression of this pathway (21, 33, 34). Peripheral ACAID signals are detectable in blood, with NKT cell, linked chemokine programs (e.g., RANTES) coordinating APC T cell co localization (35, 36). Related paradigms have been demonstrated after vitreous or subretinal antigen delivery, indicating that ocular antigen exposure outside the anterior chamber can also induce systemic deviation and tolerance (37, 38).

Complementing ACAID, ocular tissues can impose local, on demand re programming of T cells after antigen recognition within the eye. Tissue derived cues—membrane PD L1 and FasL expressed by retinal pigment epithelium (RPE), and fluid phase mediators such as TGF β2 and α MSH in ocular fluids—directly attenuate effector programs and favor regulatory outcomes (3–7). In parallel, retinal microglia, perivascular/monocyte derived macrophages, and resident dendritic cells can present retinal antigens in a tolerogenic context, supporting FOXP3^+^ T regulatory (Treg) conversion or the acquisition of anergy like transcriptional programs in uveitogenic T cells. *In vivo* profiling has now documented these regulatory/anergic signatures in T cells that have encountered antigen within the living eye, providing molecular evidence for local imprinting that complements spleen dependent deviation (20, 62–68).

Together, these systemic (ACAID type) and local, ocular routes form a coordinated framework for adaptive immune control. The former explains how intraocular antigen exposure can recalibrate systemic immunity, whereas the latter demonstrates that the retinal environment itself can re shape effector T cell fate at the site of risk. This duality underpins our view that ocular immune privilege is dynamic and, in principle, restorable when the relevant cues are re engaged (**Figure 1D**).

## 4 Privilege under siege: overview of disease mechanisms

**Figure 2:**
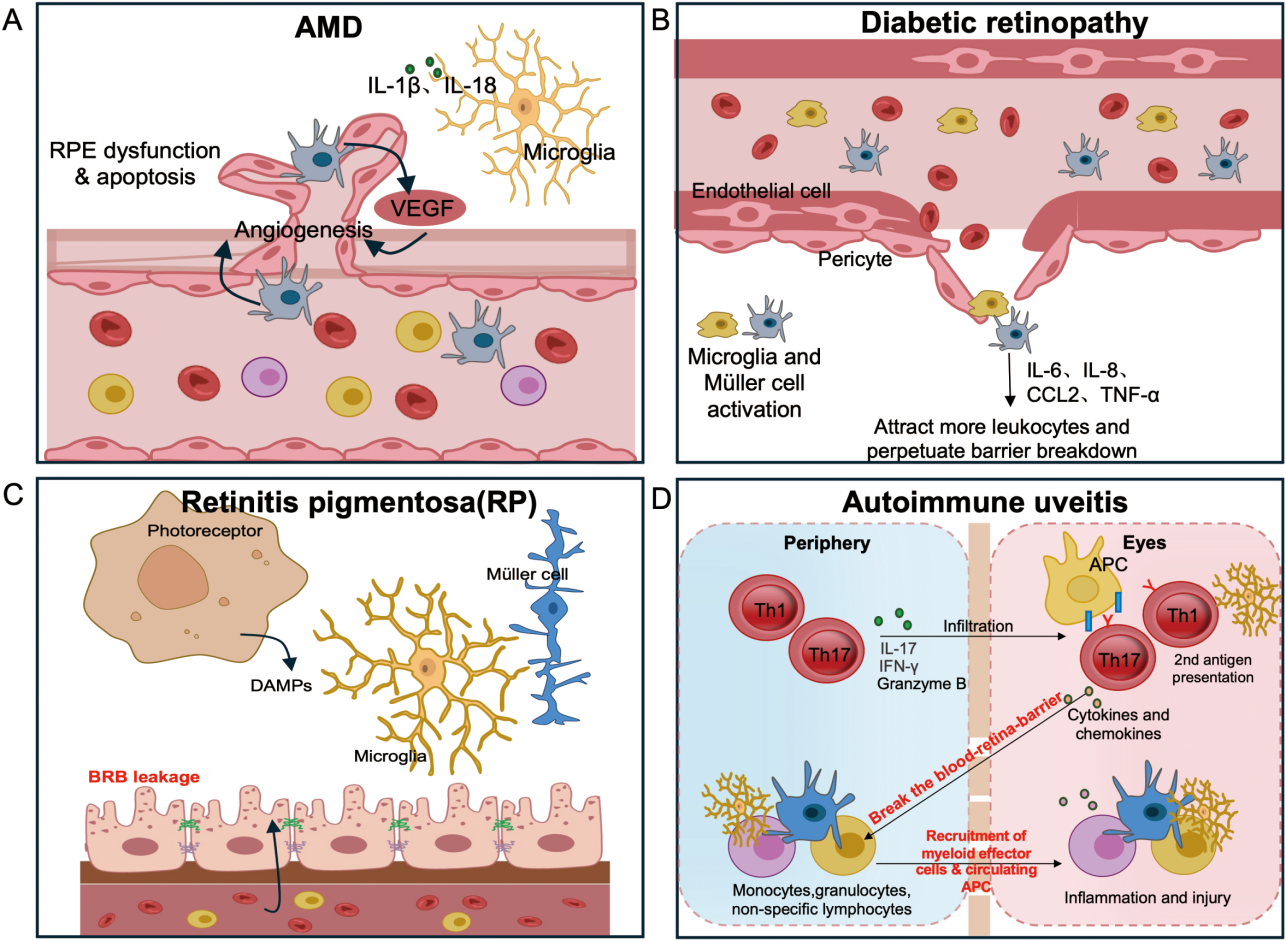
Disease-specific routes to breach retinal immune privilege.**(A)** AMD: complement-decorated drusen and subretinal myeloid accrual drive para-inflammation; outer BRB compromise emerges with choroidal neovascularization (CNV). **(B)** DR: pericyte loss and inner BRB leakage initiate leukostasis, followed by cytokine-driven edema and ischemia. **(C)** RP: degeneration-induced microglial activation leads to loss of tolerogenic cues and outer BRB integrity, permitting CCR2^+^ monocyte and T cell entry with subsequent autoreactivity. **(D)** Non-infectious (autoimmune) uveitis: Activated Th17 and Th1 cells breach the BRB and release cytokines and chemokines that recruit myeloid effectors and circulating antigen-presenting cells (APCs). Recruited APCs from the circulation provide a secondary antigen-presentation event required for full expression of spontaneous uveitis, whereas in the EAU model this role is fulfilled by resident microglia. Together these interactions amplify intraocular inflammation and tissue injury.

We use a three‐archetype framework to describe failures of immune privilege: (i) barrier failure with vascular leakage and immune cell extravasation (1, 2); (ii) innate immune dysregulation of resident microglia (often accompanied by infiltrating monocyte‐derived macrophages), reinforced by complement and inflammasomes (16, 50); and (iii) adaptive autoimmunity when retinal antigens are presented in immunogenic contexts (21, 30–34, 36–38, 69, 70). Sections 5–8 integrate these archetypes into disease‐specific narratives. Importantly, these routes rarely occur in isolation; instead, they combine in distinct proportions across retinal diseases (**Figure 2**; **Table 1**).

### 4.1 Age-related macular degeneration—innate first, barrier later

Age-related stress at the RPE–Bruch’s membrane interface, combined with complement risk alleles, establishes a para-inflammatory milieu where drusen accumulate and complement components are aberrantly deposited (16, 18). Subretinal microglia cluster around drusen and injured RPE (with monocyte-derived macrophages joining especially as disease advances), releasing IL-1β, IL-18, and proteases that amplify tissue damage (13, 18). Thus, the primary breach in early and intermediate AMD is an innate activation arising from within the eye, rather than a massive influx of circulating leukocytes (16, 18). As AMD progresses—particularly in “wet” AMD—the outer BRB becomes compromised by VEGF-driven choroidal neovascularization, which permits plasma proteins and immune cells to enter the subretinal space (49, 71, 72).

CX3CR1-dependent microglial accumulation in the subretinal space is associated with AMD pathology in both human specimens and murine models (13). Complement deposition on drusen and RPE, coupled with genetic risk alleles, implicates dysregulation of the complement cascade in AMD pathogenesis (18). Furthermore, clinical trial data show that inhibiting complement C3 (e.g. with pegcetacoplan) slows geographic atrophy progression, providing human proof-of-concept for targeting the innate complement pathway (18).

For geographic atrophy (GA), complement inhibitors reduce immune-mediated injury by dampening complement overactivation, while in neovascular AMD, anti-VEGF agents help re-seal the outer barrier and reduce inflammatory exudation (18, 49, 71, 72). Efforts to restore the retinal “glycocode” (for example, using polysialic acid to reinforce “self” signals and curb complement-mediated opsonophagocytosis) are under translational exploration (18). Clinical efficacy has been demonstrated for complement inhibition in GA and for anti-VEGF therapy in choroidal neovascularization (CNV). By contrast, glycocode restoration remains at a preclinical, experimental stage.

### 4.2 Diabetic retinopathy — barrier first with inflammatory amplification

Chronic hyperglycemia induces pericyte apoptosis and disassembly of endothelial tight junctions in retinal capillaries (inner BRB), leading to vascular leakage and capillary non-perfusion (23, 24). Loss of barrier integrity permits leukostasis: neutrophils and monocytes adhere to and clog retinal microvessels, releasing oxidants and proteases that damage the endothelium (73–75). In addition, retinal glial cells sense hyperglycemic stress and release inflammatory mediators (IL-6, IL-8, CCL2, TNF-α) that further loosen endothelial junctions and recruit leukocytes. Notably, intraocular IL-6 levels correlate with diabetic macular edema (DME) severity and directly contribute to barrier dysfunction (76–78).

Early human histopathology confirms pericyte dropout in DR patients (23). Pharmacological blockade of IL-1β (via caspase-1 inhibition) prevents capillary degeneration in diabetic animal models (75). Consistently, vitreous and aqueous humor samples from patients show elevated cytokines (IL-6, IL-8, CCL2) correlating with disease stage (76, 77, 79).

Anti-VEGF therapy reduces vascular permeability and macular edema in DR, thereby mitigating fluid leakage (19, 80). Corticosteroids help restore endothelial junction integrity and suppress leukocyte adhesion, which improves retinal swelling and inflammation (19). Meanwhile, targeting IL-6 or the NLRP3 inflammasome is being explored in preclinical studies and early clinical trials for refractory DME (81, 82). Overall, DR exemplifies a barrier-first breach that secondarily permits leukocyte-driven inflammation (22, 23, 73–79). Clinical efficacy is well established for anti-VEGF agents and corticosteroids, whereas IL-6/NLRP3-targeting strategies remain at observational and preclinical stages.

### 4.3 Retinitis pigmentosa — degeneration initiated, immune amplified

Genetic photoreceptor defects leading to cell death provide persistent damage-associated molecular patterns (DAMPs) that trigger early microglial activation and migration into the outer retina, even before overt cell loss occurs (46–49, 83). An initial compensatory rise in TGF-β1 can transiently dampen this innate activation. However, as degeneration continues, immunosuppressive cues decline and tight-junction proteins are lost. Consequently, the outer BRB loosens and allows infiltration of CCR2^+^ monocytes and T cells into the retina (26). In some patients, circulating anti-retinal antibodies arise once the barrier is breached, signaling a loss of immune tolerance (formerly immune ignorance) to retinal antigens (26, 39, 84–93).

Multiple lines of evidence support this mechanistic sequence. In RP models, microglial activation and migration clearly precede photoreceptor cell death (46–48, 83). Human RP tissues show reduced levels of immunosuppressive molecules (e.g. TGF-β1, PD-L1) and loss of junctional proteins, accompanied by increased inflammatory cytokines and immune cell infiltration (26). In a mouse RP model, recruitment of CCR2^+^ monocytes has been demonstrated, and treatment with minocycline (which suppresses microglia/monocyte activation) provides neuroprotection (27). Consistently, an open-label trial in RP patients reported functional benefits from oral minocycline (94).

Modulating microglial and monocyte activity (for example, with minocycline) and restraining complement or inflammasome activation are being investigated as adjuncts to gene or neuroprotective therapies in RP. Reinforcing innate inhibitory checkpoints (such as the CD200–CD200R pathway on microglia) is another promising strategy, though disease-specific evidence for this approach remains limited to preclinical studies (27, 60, 61, 94).

Notably, beyond its antimicrobial role, minocycline exerts pleiotropic anti-inflammatory effects centered on the microglia–monocyte axis. For instance, it inhibits the p38 MAPK/NF-κB pathway and its downstream effectors (iNOS, MMP-9, TNF-α). It also counteracts pro-inflammatory M1 polarization in microglia and attenuates CCR2-dependent monocyte recruitment *in vivo*. In retinal degeneration models, these actions translate into reduced microglial reactivity, fewer CCR2^+^ monocytes, and preserved photoreceptor cells – outcomes consistent with exploratory human findings in RP (27, 95–97).

### 4.4 Non-infectious uveitis — adaptive first, actively breaches privilege

Peripherally primed uveitogenic CD4^+^ T cells (typically Th17 and Th1 subsets) home to ocular tissues via integrin–chemokine signals and actively breach the BRB by releasing inflammatory cytokines (IL-17, IFN-γ, TNF-α) and by granzyme-mediated attack on junctional proteins (28, 98). Remarkably, only a few thousand IRBP-specific T cells are sufficient to induce fulminant uveitis in naïve mice, demonstrating the high per-cell pathogenicity of these effectors (30–32). An acute influx of neutrophils and monocytes follows this T cell invasion, leading to macrophage-mediated bystander injury and, in chronic disease, the formation of tertiary lymphoid aggregates in the eye (28, 99, 100).

These pathological mechanisms are supported by both experimental and clinical evidence. Human Th17 cells can disrupt vascular barriers in CNS models via granzyme B–mediated cleavage of tight junctions, suggesting a similar ability to compromise the BRB in uveitis (98). Consistently, adoptive transfer experiments in the EAU model (experimental autoimmune uveitis) demonstrate that antigen-specific T cells are sufficient to cause uveitic disease (30–32). Moreover, long-lived T cells appear to drive chronicity: bone marrow–resident memory CD4^+^ T cells have been shown to sustain inflammation in experimental uveitis, which may explain relapsing disease courses (99, 100).

Therapeutic interventions for non-infectious uveitis aim to control the aggressive T-cell–driven inflammation. Corticosteroids remain the first-line treatment to suppress acute ocular inflammatory flares. For steroid-refractory cases, the TNF-α inhibitor adalimumab has demonstrated an ability to induce and maintain remission (as shown in the VISUAL trials) (101). Additionally, blockade of the IL-6 receptor can benefit uveitic macular edema, reducing inflammation and vascular leakage (81). Meanwhile, tolerance-oriented approaches are emerging. In preclinical models, inducing antigen-specific regulatory T cells or B cells (Tregs/Bregs) has been shown to suppress pathogenic effector responses (102, 103). Notably, *in vivo* studies suggest that ocular tissues can imprint regulatory or anergic programs on uveitogenic T cells, implying that immune privilege is potentially restorable if these tolerogenic circuits are therapeutically engaged.

To minimize repetition, we have not summarized each disease’s details here. Instead, Sections 5–8 will open with the dominant archetype for each disorder and then develop the mechanistic narrative along with aligned translational implications.

## 5 Immune mechanisms in age-related macular degeneration

### 5.1 Early–intermediate AMD: an “endogenous” imbalance at the RPE–Bruch’s interface (drusen–complement–innate cells)

As framed in Section 4, AMD exemplifies an innate first breach—centered on complement decorated drusen and subretinal myeloid activation—with outer BRB compromise emerging mainly in neovascular stages. Hallmark drusen form between the retinal pigment epithelium (RPE) and Bruch’s membrane and contain lipids, complement components, and protein aggregates (e.g., vitronectin, amyloid-β), consistent with immunogenic/para-inflammatory foci (104–106).

The genetic underpinning of this innate dysregulation is well established, Complement-pathway variants—notably in CFH (e.g., Y402H) and C3—confer substantial AMD risk across multiple independent cohorts, implicating innate immune dysregulation in pathogenesis (104–106).

Human tissue and proteomic studies demonstrate C3b and C5b–9 decorating drusen and adjacent RPE, consistent with alternative-pathway activation at sub-RPE sites before overt clinical inflammation (18, 104–106). In the drusen-proximate niche, RPE and choroid up-regulate complement constituents and inflammatory mediators (e.g., IL-6, IL-8), establishing a low-grade para-inflammatory tone prior to ophthalmoscopically evident inflammation (18). This pattern defines early AMD as an “inside-out” disturbance: subretinal/sub-RPE innate activation predominates while BRB integrity is largely preserved; massive infiltration of circulating leukocytes is not typical at this stage (13, 18).

### 5.2 Local amplification by subretinal microglia/monocyte-derived macrophages

Under AMD-related stress, resident retinal microglia migrate from the inner retina toward the subretinal interface, while monocyte-derived macrophages infiltrate from the choroid through the compromised RPE or Bruch’s membrane. They cluster around drusen deposits or sites of RPE injury and release IL-1β, proteases, and complement proteins that can promote RPE dysfunction and contribute to atrophy (13, 55).

Apolipoprotein E (ApoE) supports the long-term survival of these subretinal mononuclear phagocytes, sustaining a chronic inflammatory microenvironment and emphasizing the intersection between lipid metabolism and innate immune pathways in AMD (55).

As a result, early AMD represents a para-inflammatory state dominated by innate immune activity, with inflammation compartmentalized within the subretinal and sub-RPE spaces rather than involving a generalized leukocyte influx (13, 18).

### 5.3 Advanced AMD bifurcation: geographic atrophy *vs* neovascular AMD

#### 5.3.1 Geographic atrophy

GA margins show dense rims composed of both activated microglia and infiltrating monocyte−derived macrophages, paralleling the expansion of atrophic areas (13). Clinical trial evidence supports a mechanistic role for complement activation in GA progression: inhibition of complement component C3 with pegcetacoplan slowed GA lesion growth in a randomized study, with subsequent trials leading to regulatory approval and validating innate pathway suppression as a tissue-preserving strategy in AMD (18) (104–106). Similarly, avacincaptad pegol, an RNA aptamer targeting complement C5, reduced GA enlargement in the GATHER2 trial and received FDA approval; monthly dosing slowed lesion expansion compared with sham at 12 months, with durable effects in longer-term analyses. Updated labeling in 2025 permits flexible treatment durations. Together with C3 inhibition data from the OAKS and DERBY trials, these results establish complement modulation as a clinically validated approach to lowering the innate immune set point and slowing tissue loss in AMD (107–110).

#### 5.3.2 Neovascular AMD

In neovascular AMD, hypoxia resulting from choriocapillaris rarefaction induces VEGF-driven choroidal neovascularization that breaches the outer blood–retina barrier at the level of RPE tight junctions, allowing plasma proteins and immune components to enter the subretinal compartment (49, 71, 72, 111). The anti-VEGF treatment landscape has advanced beyond first-generation agents such as ranibizumab, bevacizumab, and aflibercept 2 mg to newer, longer-acting formulations and dual-target strategies. High-dose aflibercept 8 mg (Eylea HD) enables dosing intervals of 8–16 weeks following three monthly injections across nAMD, DME, and DR indications while maintaining visual and anatomical outcomes, as reflected in the FDA label. Faricimab (Vabysmo), a bispecific Ang-2/VEGF-A inhibitor, demonstrated non-inferior visual outcomes compared with aflibercept in the TENAYA and LUCERNE trials, with dosing intervals of up to 16 weeks in treat-and-extend regimens. Collectively, these agents restore the integrity of the outer blood–retina barrier, reduce vascular leakage, and secondarily limit inflammatory ingress, while offering materially improved treatment durability (104–106, 112–116).

### 5.4 Beyond complement: inflammasome (NLRP3) and the glycocode (sialylation) checkpoint

Oxidative and RNA-processing stress within the retinal pigment epithelium (RPE)—for example, loss of DICER1 leading to Alu RNA accumulation—activates the NLRP3 inflammasome, which promotes IL-1β and IL-18 maturation and induces RPE degeneration. This sequence of events constitutes a complement-independent inflammatory amplifier in AMD (104–106). In addition, drusen constituents such as amyloid-β likewise activate NLRP3 in retinal innate cells, creating complement–inflammasome crosstalk (104–106).

Terminal sialic acids on cell surfaces encode a “self” glycocode that restrains complement deposition and microglial phagocytosis; with aging, declining retinal sialylation likely lifts this brake (18). In mice, genetic hyposialylation produces C3-driven retinal inflammation with AMD-like features, whereas intravitreal polysialic acid (polySia) reduces mononuclear phagocyte activation and complement deposition, preserving retina. Early clinical investigations of polySia therapy for geographic atrophy have begun, highlighting sialylation control as a potential strategy for mitigating complement−mediated injury in AMD (18).

### 5.5 Synthesis and translational implications

AMD exemplifies a progressive, innate−driven erosion of ocular immune privilege in which complement activation, microglial and monocyte−derived macrophage recruitment, and subsequent RPE injury form a coherent pathogenic axis. As the disease advances, breakdown of the outer blood–retina barrier becomes prominent, particularly in neovascular AMD, further permitting peripheral immune components to access the subretinal space and reinforce chronic inflammation (13, 18, 49, 71, 72).

Clinical validation of immune modulation underscores the therapeutic relevance of this axis. Inhibition of complement component C3 slows the expansion of geographic atrophy, supporting the principle of lowering innate immune activity to preserve retinal tissue. Likewise, anti−VEGF therapy reduces vascular leakage and angiogenesis and, by restoring barrier integrity, indirectly limits inflammatory ingress (18, 104–106).

Although AMD remains largely an innate disorder, ocular tissues retain the intrinsic ability to imprint regulatory or anergic programs on infiltrating T cells. This capacity highlights that immune privilege within the eye is not fixed but dynamically adaptable and, in principle, restorable. Recognizing this reinforces forward−looking therapeutic directions focused on checkpoint stabilization and tolerance−oriented repair—concepts that extend beyond innate modulation toward re−establishment of immune equilibrium in degenerative retinal disease (104–106).

Evidence: Observational human (tissue, fluids, proteomics, genetics, and drusen composition); Preclinical (mechanistic support, causality, and efficacy in models aligning with human signals); Interventional human (complement inhibition for GA, e.g., pegcetacoplan; anti-VEGF for CNV supporting barrier failure); Early translational/clinical exploration (ongoing for tolerance/checkpoint concepts and human translation).

## 6 Immune mechanisms in diabetic retinopathy

### 6.1 Initiation: metabolic injury of the neurovascular unit and inner BRB failure

Diabetic retinopathy exemplifies a disease that originates with barrier failure: weakening of the inner blood–retina barrier (BRB) and ensuing leukostasis mark the onset of pathology, while cytokine-driven inflammation subsequently amplifies edema and ischemia. Chronic hyperglycemia initiates a reproducible pattern of microangiopathy within retinal capillaries, characterized by pericyte apoptosis, basement-membrane thickening, endothelial cell loss, and formation of microaneurysms, all culminating in progressive inner BRB weakening (22, 23, 117–122). Pericytes are particularly susceptible to injury—appearing as “pericyte ghosts” in histological sections—and their depletion, coupled with VEGF up-regulation, leads to down-regulation of tight-junction proteins such as occludin and ZO-1, thereby destabilizing endothelial barriers (22, 23). Functionally, retinal vascular permeability increases very early in the disease course, often preceding clinically recognizable retinopathy, consistent with the concept that diabetic retinopathy begins as a vascular breach that permits inflammatory amplification (19).

### 6.2 After the breach: leukocytosis, capillary occlusion, and ischemic amplification

With compromise of the inner blood–retina barrier, circulating leukocytes and plasma−derived factors gain access to the retinal parenchyma. A defining feature of early diabetic retinopathy is leukostasis—abnormal adhesion of neutrophils and monocytes to the capillary endothelium—which contributes to capillary non−perfusion and focal ischemia (73–75, 123, 124). These adherent leukocytes release reactive oxygen species and proteolytic enzymes that damage endothelial cells, further propagating BRB disruption and extending the area of vascular compromise (73–75, 123, 124).

In parallel, the intraocular cytokine milieu in diabetic retinopathy consistently shows elevated levels of IL−6, IL−8 (CXCL8), and CCL2 (MCP−1) (76, 77, 79). Mechanistically, IL−6 signaling up−regulates endothelial adhesion molecules such as ICAM−1 and VCAM−1, while diminishing the barrier−supportive functions of retinal glia and endothelium, thereby promoting leukocyte adhesion and paracellular permeability. Elevated ocular IL−6 correlates with greater severity of diabetic macular edema, and experimental IL−6 blockade reduces vascular leakage in preclinical models, highlighting its contributory role in barrier failure and inflammatory amplification (78, 81).

### 6.3 Resident innate cells in a diabetic retina: microglial activation and inflammasome signaling

Retinal microglia in experimental diabetes undergo a transition from surveillant to activated states, up-regulating inducible nitric oxide synthase (iNOS) and pro-inflammatory cytokines while proliferating within the ganglion cell and plexiform layers (122). This activation coincides with neuronal stress signals; photoreceptors exposed to hyperglycemia produce inflammatory mediators that can induce endothelial apoptosis, illustrating the bidirectional neurovascular crosstalk that directly links neuronal distress to microvascular injury (88). Among the inflammatory mediators implicated, two cytokine axes-TNF-α and IL-1β-are particularly prominent. TNF-α is consistently elevated in diabetic vitreous, especially in proliferative diabetic retinopathy, and can trigger endothelial cell apoptosis (125). IL-1β, generated through caspase-1 activation, contributes to capillary degeneration, and experimental inhibition of caspase-1 or IL-1β signaling prevents capillary loss in diabetic and galactosemic models (75). Upstream, high glucose and oxidized lipid stress activate the NLRP3 inflammasome within retinal microglia and infiltrating monocyte−derived macrophages, producing IL−1β and IL−18 that further increase vascular permeability and promote pericyte death. Pharmacologic inhibition of NLRP3 with the small−molecule inhibitor MCC950 reduces retinal inflammation and vascular leakage in diabetic models, underscoring inflammasome activation as a pivotal amplifier of neurovascular injury (82).

### 6.4 Progression to proliferative disease: hypoxia → VEGF → pathological angiogenesis

Capillary closure and ensuing retinal ischemia lead to up−regulation of vascular endothelial growth factor (VEGF), which drives pathological neovascularization on the retinal surface in proliferative diabetic retinopathy. The newly formed vessels are structurally fragile and prone to hemorrhage, compounding the risk of vision−threatening events (19). In addition to its angiogenic role, VEGF is a potent mediator of vascular permeability, amplifying macular oedema; therapeutic suppression of VEGF not only reduces leakage and edema but can also indirectly attenuate inflammatory activity by alleviating hypoxia within the retinal microenvironment (80, 126). Nevertheless, 50% of DME patients show suboptimal anatomical or visual responses to anti−VEGF therapy, indicating that VEGF−independent inflammatory pathways sustain disease activity in a substantial subset of individuals (19, 80).

### 6.5 Neurodegeneration within DR: early neuronal dysfunction and immune cross-talk

Diabetic retinopathy is not purely a microvascular disorder; neuronal dysfunction involving amacrine and ganglion cells can often be detected by electroretinography and visual function testing before classic microvascular lesions become apparent (127–131). Within the diabetic retina, activated microglia and reactive Müller glia release excess glutamate and reactive oxygen species, intensifying neuronal stress and injury (122). In parallel, photoreceptor−derived mediators aggravate endothelial damage and contribute to capillary loss, further interlinking neural and vascular pathology (88). With the BRB compromised, systemic low-grade inflammation typical of diabetes more readily propagates into retinal tissue, fueling a self-reinforcing inflammatory loop (73).

### 6.6 Therapeutic implications: reseal the barrier and lower the inflammatory set-point

Optimized metabolic control of glycaemia, blood pressure, and lipids remains the foundation of diabetic retinopathy management. Anti−VEGF agents such as aflibercept, bevacizumab, and ranibizumab effectively reduce diabetic macular oedema and pathological neovascularization, yet a considerable proportion of patients show incomplete anatomical or functional responses (80). Intravitreal corticosteroids, including dexamethasone and fluocinolone implants, enhance tight−junction integrity, suppress ICAM−1 and VEGF expression, and diminish leukocyte recruitment, thereby improving diabetic macular oedema, although their use is tempered by the risks of cataract formation and intraocular pressure elevation (19). Given the established association between IL−6 levels and DME severity, blockade of the IL−6 receptor with tocilizumab has shown encouraging signals in small clinical series and early−phase trials of refractory disease (81).

Targeting innate inflammatory pathways provides an additional therapeutic dimension. Inhibition of caspase−1 or IL−1β prevents capillary degeneration in experimental models, while pharmacologic blockade of the NLRP3 inflammasome with MCC950 reduces vascular leakage and retinal inflammation *in vivo* (75, 82). TNF−α neutralization is mechanistically reasonable given its elevated vitreous levels in advanced retinopathy; off−label use in severe, refractory cases has been reported, though it is not standard of care (19, 125). Chemokine−mediated monocyte recruitment also contributes to disease propagation, and blockade of the CCR2/CCL2 axis shows preclinical promise. However, meaningful clinical efficacy in diabetic macular oedema remains to be established, and such approaches are not currently part of routine management (19, 76, 77, 79).

DR typifies a barrier-first breach that then permits leukocyte-driven inflammation, effective therapy therefore couples barrier stabilization with targeted suppression of inflammatory cascades (19, 22, 23, 73–80). As a unifying lens, the concept that ocular immune privilege is dynamic and, in principle, restorable—through attenuation of innate immune “noise” and re−establishment of local regulatory cues—provides a useful framework as immunomodulatory strategies in diabetic retinopathy continue to evolve. Clinical efficacy is firmly established for anti−VEGF agents and corticosteroid implants, whereas additional immunomodulatory approaches remain at the observational or preclinical stage and require prospective validation before entering routine practice.

Evidence: Human histopathology and clinical imaging, with mechanistic confirmation from animal and ex vivo studies; Observational human (fluid cytokines, functional testing, and tissue associations) with preclinical support for causal pathways; Interventional human evidence for anti-VEGF/steroid efficacy, tempered by non-responder populations and observational data supporting residual inflammation; Preclinical and observational signals for IL-6, TNF-α, inflammasome, and CCR2–CCL2 pathways, with early clinical exploration for IL-6R blockade.

## 7 Immune mechanisms in inherited retinal degenerations (retinitis pigmentosa)

### 7.1 Natural history and immune context

Inherited retinal degenerations (exemplified by RP) begin with degeneration triggered innate activation and can evolve toward barrier loosening with adaptive footprints in later stages. Retinitis pigmentosa (RP) comprises >100 genetic disorders that primarily kill rod photoreceptors and secondarily cones, typically beginning in youth or early adulthood (26). Over years to decades, the normally immune−privileged retinal environment undergoes gradual immunologic remodeling, transitioning from subtle para−inflammatory changes to overt barrier compromise and the emergence of adaptive immune responses (26).

### 7.2 Early innate sensing: microglia engage before overt cell loss

Across multiple models of retinitis pigmentosa, microglial activation and migration into the outer retina occur before, or concurrently with, photoreceptor death. This temporal relationship indicates that dying or stressed photoreceptors release danger−associated cues that recruit and activate resident retinal myeloid cells (46–49, 83). Once activated, microglia acquire amoeboid morphologies within the photoreceptor layers and closely engage with degenerating rods (46–49, 83). Early compensatory regulatory responses are also evident: retinal TGF−β1 levels can rise transiently during the initial stages of degeneration, reflecting a local attempt to restrain microglial reactivity and promote controlled debris removal (26, 53, 58, 132).

### 7.3 “Privilege under pressure”: loss of local immunoregulation and BRB loosening

As photoreceptor death persists, the continual release of intracellular danger−associated molecular patterns (DAMPs) sustains activation of retinal microglia and infiltrating macrophages. In both patients and mouse models of retinitis pigmentosa, anti−inflammatory programs progressively diminish while pro−inflammatory cytokine expression rises: levels of TGF−β1 and PD−L1 decline in degenerating retina, and tight−junction proteins within the retinal pigment epithelium and vascular endothelium are downregulated, signaling breakdown of both the outer and inner blood–retina barriers (26). Functionally, this marks a transition from compartmentalized innate activation to a system-exposed retina, enabling ingress of blood-borne mediators and cells (26).

### 7.4 Emergent adaptive autoimmunity: antibodies and T cell footprints

As BRB integrity falters, retinal antigens (opsins, arrestin, recoverin, enolase) can reach lymphoid organs and be presented to T cells. Circulating anti-retinal autoantibodies occur in a subset of RP patients, signaling loss of immune ignorance/tolerance (39, 84–93). Correspondingly, in murine models of RP, T cells become detectable within late−stage retinas, consistent with secondary adaptive immune infiltration following barrier failure (26).

### 7.5 Myeloid crosstalk and recruitment from blood

Beyond resident microglia, CCR2^+^ monocytes are recruited from the circulation (notably after barrier loosening) and differentiate into inflammatory macrophages that amplify cytokine/ROS production and tissue injury (27). In the absence of appropriate regulatory or reparative polarization, recruited macrophages remain pro−inflammatory and operate in a feed−forward loop with resident microglia (27).

### 7.6 Maladaptive phagocytosis and cone jeopardy

In degenerating retinas, microglia can shift from a protective role in clearing apoptotic corpses to a maladaptive state in which they phagocytose stressed but still viable photoreceptors, thereby accelerating neuronal loss (46–49, 51). In retinitis pigmentosa models, CX3CL1-CX3CR1 signaling constrains this aberrant phagocytic activation; disruption of the pathway aggravates degeneration, whereas augmentation of soluble CX3CL1 preserves cone survival and visual function *in vivo* (51, 59). Spatial single-cell analyses further demonstrate location-specific microglial states across retinal layers during both homeostasis and degeneration, underscoring the context-dependent nature of microglia-neuron interactions (50).

### 7.7 Mechanistic synthesis

Phase I (intrinsic degeneration). Gene-driven photoreceptor death → local microglial activation and transient tolerogenic cues (TGF-β1) (26, 46–49, 51, 58, 132).

Phase II (immune-accelerated degeneration). Declining immunosuppressive checkpoints (e.g., PD-L1, TGF-β1) + BRB loosening → CCR2^+^ monocyte and T cell access → anti-retinal antibodies in subsets → sustained inflammation that exceeds the original genetic injury (26, 27, 39, 84–90, 92).

### 7.8 Therapeutic implications: lowering the innate set-point and rebuilding restraint

Microglia/monocyte modulation. In murine models of retinitis pigmentosa, minocycline attenuates microglial activation and reduces CCR2^+^ monocyte recruitment, thereby preserving photoreceptors and maintaining visual function; open-label clinical observations in RP patients report measurable visual function benefits over 6-12 months (27, 94).

Innate checkpoints. The CX3CL1-CX3CR1 axis represents a druggable target: adeno-associated viral delivery of soluble CX3CL1 improves cone survival and function in RP models (60). Similarly, engagement of the CD200-CD200R pathway restrains myeloid activation; agonism of CD200R reduces myeloid-driven damage in ocular degeneration models, supporting the concept of checkpoint reinforcement for chronic retinal disease (8, 10, 60, 61).

Restoring local immunoregulation. TGF-β signaling is essential for maintaining retinal microglia in a restrained state; loss of microglial TGF-β signaling precipitates degeneration and worsens neovascular sequelae, highlighting TGF-β based regulatory circuits as potential therapeutic levers (53, 58).

Adaptive containment and privilege restoration. Although RP is not primarily autoimmune, late-stage adaptive immune involvement can arise when ocular immune privilege collapses. Conceptually, strategies that re-impose tolerance, such as promoting local regulatory programs-are consistent with *in vivo* evidence that the eye can imprint regulatory or anergic gene signatures on pathogenic T cells (62, 68, 133, 134).

What to avoid/what remains uncertain. Broad systemic immunosuppression (e.g., chronic corticosteroids) has limited utility in RP due to unfavorable risk–benefit profiles. More targeted approaches, such as modulation of myeloid checkpoints, fine−tuning of IL−1/TNF signaling, and selective chemokine axis intervention, warrant stage−specific evaluation in appropriate models. The contribution of complement pathways in RP appears model−dependent; while opsonization may mark stressed photoreceptors, robust patient−level evidence for complement blockade efficacy in RP is lacking, in contrast to its established role in geographic atrophy secondary to AMD.

Evidence: Observational human (natural history, tissue/fluids, autoantibodies, and variability) with preclinical support for immune remodeling, T cell entry, and mechanistic corroboration; Preclinical evidence (temporal sequence, cellular states, spatial omics, and strong mechanisms) showing alignment and concordance with human data, though often with limited or exploratory human support and causal weighting yet to be defined; Preclinical frameworks for tolerance-oriented approaches and novel clinical strategies are established, but human translation and applicability remain pending or under exploration; Preclinical and observational systems both demonstrate significant heterogeneity.

## 8 Immune mechanisms in autoimmune uveitis

### 8.1 Definition, scope, and models

Non-infectious uveitis represents the adaptive dominant extreme: antigen specific Th17/Th1 cells actively breach the BRB and recruit innate effectors. A major subset of non-infectious uveitis is autoimmune, targeting retina and/or uvea and representing an acute or subacute collapse of ocular immune privilege driven by aberrant adaptive immunity rather than secondary responses to tissue stress (20). Classic entities include sympathetic ophthalmia (trauma-triggered, bilateral autoimmunity) and birdshot chorioretinopathy (HLA-A29-associated T cell disease) (20). Mechanistically, experimental autoimmune uveitis (EAU)—induced by immunization with retinal proteins (IRBP, S-antigen) or by adoptive transfer of primed T cells—demonstrates antigen-specific T cell sufficiency for intraocular inflammation *in vivo (*30–32).

Complementing immunization-induced EAU, the IRBP-specific TCR-transgenic R161H mouse develops spontaneous, adjuvant-free uveitis and thus offers face validity for non-infectious autoimmunity (30). Mechanistically, this spontaneous model revealed that retina-specific T cells can be activated by commensal microbiota, and it helped delineate the distinct contributions of circulating (bone marrow–derived) versus resident retinal antigen-presenting cells (APCs) to disease initiation and persistence (30, 62, 64, 135–138).

### 8.2 Peripheral priming and the uveitogenic effector pool

CD4^+^ T-helper cells of the Th17 and Th1 lineages are the principal orchestrators of uveitic pathology. These uveitogenic T cells are initially primed in secondary lymphoid organs (peripheral lymph nodes). In induced EAU, naive T cells recognizing an ocular self-antigen become activated in the draining lymph nodes near the site of immunization. By contrast, in spontaneous autoimmune uveitis (e.g. the R161H model), retina-specific T cells can be activated within gut-associated lymphoid tissue by commensal microbial antigens via molecular mimicry of ocular self-antigens. Once primed, the activated T cells differentiate, upregulate homing receptors and effector programs, enter the circulation, and ultimately home to the eye (30–32). Th17 biology is pivotal in tissue-targeted autoimmunity across organ systems: notably, IL-23, rather than IL-12, is required to sustain encephalitogenic (autoaggressive) Th17 responses *in vivo* – an insight that applies to ocular models as well (139). Th1 cells (characterized by IFN-γ production) also contribute to pathology by upregulating MHC class II and adhesion molecules, thereby enhancing local antigen presentation and activating macrophages (20).

### 8.3 Active breach of the blood–retinal barrier

Uveitic BRB failure is active, driven by effector T cells, rather than merely passive leak. Human Th17 cells can disrupt CNS endothelial barriers, a paradigm applicable to the BRB (98). Mechanistically, granzyme B released extracellularly by activated lymphocytes can cleave junctional proteins (e.g., occludin) and matrix components, compromising barrier integrity (140). In ocular settings, Th17-derived mediators (IL 17A/IL 22) and granzyme programs are implicated in early barrier stripping that facilitates cellular influx (28, 98). In adoptive transfer EAU, the pathogenic T cell threshold can be extremely low: as few as 40 activated IRBP-specific CD4^+^ T cells can precipitate uveitis in Lewis rats, whereas on the order of 10³-10^4^ cells are typically required to induce disease in mice (30–32, 141). These observations underscore the high per-cell pathogenic potential of uveitogenic T cells, establishing that even a very small number of appropriately primed T cells is sufficient to actively breach ocular privilege.

### 8.4 Effector cascade inside the eye: neutrophils, monocytes, macrophages

Following T cell entry, wave-like recruitment of neutrophils (early) and monocytes (later) produces a fulminant inflammatory milieu: macrophages activated by IFNγ/IL 17/TNFα/IL 1 release ROS, proteases and cytokines, driving bystander retinal injury (photoreceptor and inner retinal damage) (20, 28). Intravital imaging in rodent EAU visualizes leukocyte rolling, adhesion, and transmigration across retinal vessels contemporaneous with early clinical signs (20). Microglia, beyond being bystanders, can initiate and amplify neuroinflammation in ocular autoimmunity, linking resident innate circuits to T cell pathology (142). The precise role of microglia is still a matter of active investigation, but emerging evidence indicates that beyond the initial priming of T cells in peripheral lymphoid organs, a second antigen-presenting event within the retina is required for full development of autoimmune retinitis. In EAU models, resident microglia appear to serve as the critical APCs that restimulate infiltrating effector T cells inside the eye, whereas in spontaneous autoimmune uveitis models this secondary antigen presentation is mediated by circulating APCs (e.g., dendritic cells) that enter the retina (64, 68, 138, 143). These findings underscore the necessity of local (intraocular) antigen presentation in unleashing the effector cascade, with either microglia or infiltrating APCs (depending on the context) fulfilling the requisite second signal for robust intraocular inflammation.

### 8.5 Chronicity, relapse, and tissue “memory”

A hallmark of chronic uveitis is the persistence of tissue-tropic memory T cells. Autoreactive memory CD4^+^ cells can reside in bone marrow to perpetuate or rekindle disease, providing a reservoir that sustains chronicity/relapse (99). Long-lived autoreactive memory populations sustaining chronic retinopathy are similarly demonstrated (101). Local tertiary lymphoid-like aggregates and *in situ* antigen presentation have been described in prolonged disease, reflecting a self-sustained intraocular immune niche at odds with immune privilege (100). Notably, experimental rat uveitis models developed by Wildner and colleagues demonstrate that the disease course (monophasic *vs*. relapsing-remitting) is largely determined by the autoantigen used for immunization, providing insight into immune mechanisms that drive chronicity and relapse in uveitis (20, 144–146).

### 8.6 Regulation, resolution, and the possibility of privilege restoration

Under physiologic privilege, regulatory T cells (Tregs) and regulatory B cells (Bregs) restrain pathogenic immunity. Importantly, the eye can actively induce regulatory T cells: Gregerson and McPherson have shown that antigen-specific Foxp3^+^ Tregs are generated locally when peripheral T cells encounter retinal antigens within the eye, highlighting a mechanism by which the retina reinforces immune privilege *in situ* (62, 66, 147). In human uveitis, however, Tregs can be numerically or functionally inadequate, and an imbalance favoring effectors over Tregs correlates with disease activity (20). In preclinical models, expansion or adoptive transfer of antigen-experienced Tregs suppresses ocular inflammation and arrests progressive retinal damage, demonstrating the therapeutic potential of restoring regulatory balance (103). Bregs induced by IL 35 suppress Th17/Th1 responses and confer resistance to EAU upon adoptive transfer (proof of concept for tolerance-inducing cell therapy) (102). Critically, *in vivo* profiling demonstrates that the living eye can imprint regulatory/anergic programs on uveitogenic T cells (68, 133), supporting the concept that ocular immune privilege is active-and, in principle, restorable.

### 8.7 Therapeutic implications: from flare control to durable immune re-education

Corticosteroids administered locally or systemically remain the first-line therapy for acute uveitic flares, acting to rapidly suppress T cell and myeloid effector programs (20).

For longer-term, steroid-sparing disease control, blockade of TNF-α (e.g., with adalimumab) prolongs remission and reduces relapse frequency in randomized trials, findings that underpin its regulatory approval for refractory non-infectious uveitis (101). Inhibiting IL-6 receptor signaling with tocilizumab yields particular benefit in uveitic macular edema and refractory disease, consistent with IL-6’s role in promoting barrier disruption (81).

More mechanistically targeted and tolerance-oriented strategies are under exploration: clinical experience with IL-17 pathway inhibitors has been mixed-reflecting Th17’s potent initiating function and network redundancy-prompting interest in alternative approaches such as co-stimulation blockade or modulation of adhesion pathways to limit T cell trafficking (20). Antigen-specific tolerance induction and Treg-based cellular therapies are also in active preclinical development; adoptive transfer of Tregs confers structural and functional protection in experimental models (103).

Integrative strategies that simultaneously attenuate effector inflammation (e.g., via TNF−α or IL−6 inhibition), reinforce barrier stability, and restore regulatory dominance through Treg/Breg populations or local tolerogenic cues are mutually reinforcing rather than exclusive, and they align with *in vivo* evidence that the ocular environment retains intrinsic capacity for tolerogenic re−imprinting.

### 8.8 Synthesis

Autoimmune uveitis represents the adaptive immune extreme of retinal inflammation, in which antigen-specific Th17 and Th1 cells actively breach the blood–retina barrier, recruit innate effectors, and can devastate retinal tissue within days to weeks (28, 30–32, 98). Chronicity arises from durable autoreactive memory reservoirs and the establishment of local immune niches that sustain intraocular inflammation (20, 99, 100). Encouragingly, advances in targeted immunotherapy-exemplified by randomized trials of adalimumab-have improved clinical outcomes, while emerging tolerogenic approaches based on regulatory T and B cells, as well as ocularly induced anergy and regulatory re-programming, offer a rational path toward durable remission and restoration of immune privilege (101–103).

Evidence: Preclinical models establish causality and define mechanisms (e.g., Treg/Breg sufficiency, cellular imprinting, trafficking); observational human data define clinical phenotypes, cytokine milieus, and immunological profiles (e.g., Treg imbalance); Preclinical paradigms (EAU/EAE) and imaging studies show concordance with human data, though the precise contribution to patient relapse kinetics remains under study; Interventional human evidence for anti-TNF and IL-6R therapies is supported by observational biomarkers and preclinical exploration of tolerance-oriented strategies, including cellular re-education.

## 9 Therapeutic implications and future directions

### 9.1 A cross-disease principle: re-sealing the blood–retinal barriers

Across AMD, DR, RP and uveitis, barrier integrity is a final common pathway for preserving immune privilege and limiting inflammatory flux. In practice, agents that reduce permeability and stabilize endothelium/RPE improve outcomes: anti-VEGF reduces leakage in DR/DME and nAMD in randomized trials (71, 72, 80, 111), while corticosteroids (e.g., dexamethasone/fluocinolone implants) help restore tight-junction proteins and damp leukocyte adhesion, improving DME albeit with IOP/cataract risks (19). Beyond these staples, glia-to-endothelium signaling offers a barrier-protective target; experimental Wnt/β-catenin modulation in Müller cells ameliorates ischemia-induced neovascularization, implying that strengthening neurovascular crosstalk can resist BRB failure (57). In parallel, adhesion-axis interventions informed by leukocytosis biology (e.g., ICAM-1/LFA-1, α4-integrin/VCAM-1) reduce leukocyte transmigration and BRB breakdown *in vivo*, supporting trafficking-based barrier protection in inflammatory retinopathies. Concurrently, interval biologics restore barrier integrity and reduce inflammatory flux with lower treatment burden (104–106, 112–116).

### 9.2 Mechanism-aligned immunotherapy: match the drug to the dominant driver

AMD (innate-dominant). Disease-relevant proof comes from complement inhibition: a C3 inhibitor (pegcetacoplan) and C5 inhibitor (Avacincaptad pegol) slowed GA enlargement in a randomized trial, validating innate-pathway suppression as tissue-preserving therapy (104, 108–110). Upstream triggers beyond complement—e.g., RPE inflammasome (NLRP3) activation—provide additional targets. A translationally novel axis is the “glycocode”: age-related loss of sialylation lifts complement brakes, whereas polysialic acid (polySia) reduces mononuclear phagocyte activation and complement deposition *in vivo*, suggesting checkpoint-restoring strategies (18).

DR (barrier-first with inflammatory amplification). Cytokine/innate hubs can be productively targeted: caspase-1/IL-1β inhibition prevents capillary degeneration in diabetic/galactosemic models (75); NLRP3 inhibition (MCC950) reduces retinal leakage and inflammation *in vivo* (34); and IL-6R blockade (tocilizumab) shows signals in refractory DME, consistent with ocular IL-6 elevations and its barrier-disruptive actions (76–79, 81). Anti-VEGF remains foundational, yet non-responders highlight VEGF-independent inflammation calling for combined, mechanism-guided therapy (19, 80).

RP (degeneration-initiated, immune-amplified). Microglia/CCR2^+^ monocyte modulation is mechanistically coherent: minocycline reduces CCR2^+^ recruitment, attenuates microglial activation and preserves photoreceptors in RP mice, with open-label human signals (27, 94). AAV-soluble CX3CL1 gene therapy improves cone survival/function—targeted augmentation of a physiological myeloid checkpoint (59). TGF-β signaling in retinal microglia is required for restraint; its loss induces degeneration, positioning TGF-β circuits as restorative levers (53).

Autoimmune uveitis (adaptive-dominant). TNF-α neutralization (adalimumab) prolongs remission in randomized trials and is approved for refractory disease (101); IL-6R blockade (tocilizumab) benefits uveitic macular oedema/refractory inflammation (81). Given Th17/Th1 dominance, co-stimulation and trafficking axes remain logical complements to cytokine blockade, especially in relapsing phenotypes (20).

### 9.3 From flare control to privilege restoration: tolerogenic immunotherapy

The privilege paradigm suggests that durable remission will require re-imposing tolerance, not only suppressing effector pathways. Classical ACAID/VCAID experiments show ocular antigens can elicit systemic deviation toward regulation (21, 33–38, 69, 70). Preclinically, adoptive transfer of antigen-experienced Tregs arrests retinal damage in spontaneous uveitis (103), while IL-35-induced Bregs suppress Th17/Th1 programs and confer resistance to EAU upon transfer (102). Crucially, *in vivo* single-cell/transcriptomic profiling demonstrates that the living eye can imprint regulatory/anergic gene signatures on uveitogenic T cells, indicating that ocular privilege is active and, in principle, restorable—a mechanistic foundation for tolerance-oriented therapies.

### 9.4 Microglia: deplete, deter, or reprograms?

CSF1R-mediated microglial depletion blunts ocular inflammation and limits myeloid-driven pathology in models (EAU, CNV) (142, 148), yet spatial single-cell analyses reveal location-specific microglial states with context-dependent protective roles, arguing for precision reprogramming rather than indiscriminate ablation (50). Candidate re-educators include CD200R agonism (myeloid checkpoint reinforcement) (60, 61), CX3CL1 supplementation (AAV-sCX3CL1) for photoreceptor preservation (59), and trophic immune hybrids such as IGF-1, which shifts microglia toward neuroprotection and preserves photoreceptors/RGCs *in vivo* (model- and dose-dependent caveats apply) (149–151).

### 9.5 Delivery platforms and on-site immunomodulation

The eye’s accessibility enables local, sustained immunotherapy: intravitreal steroid implants deliver durable barrier/anti-inflammatory benefits (19); AAV vectors can express immune modulators in retinal cells (e.g., soluble CX3CL1), achieving long-lived intraocular checkpoint augmentation (59). Such on-site expression minimizes systemic exposure while directly lowering the intraocular inflammatory set-point.

### 9.6 Toward precision immunotherapy

Mechanism-aligned stratification is already feasible. In DR/DME, aqueous/vitreous cytokine profiles (e.g., IL-6/IL-8/CCL2) correlate with disease activity and can inform adjunct choices (76–79). In AMD, complement genetics (CFH, C3, CFI) not only explain susceptibility but may predict who benefits most from complement-targeting strategies. Across diseases, response heterogeneity to anti-VEGF or immunobiologics underscores the need for molecular phenotyping to allocate complement inhibitors, cytokine blockers, myeloid checkpoint enhancers or tolerance-inducing regimens (80, 101).

### 9.7 Synthesis

**Figure 3:**
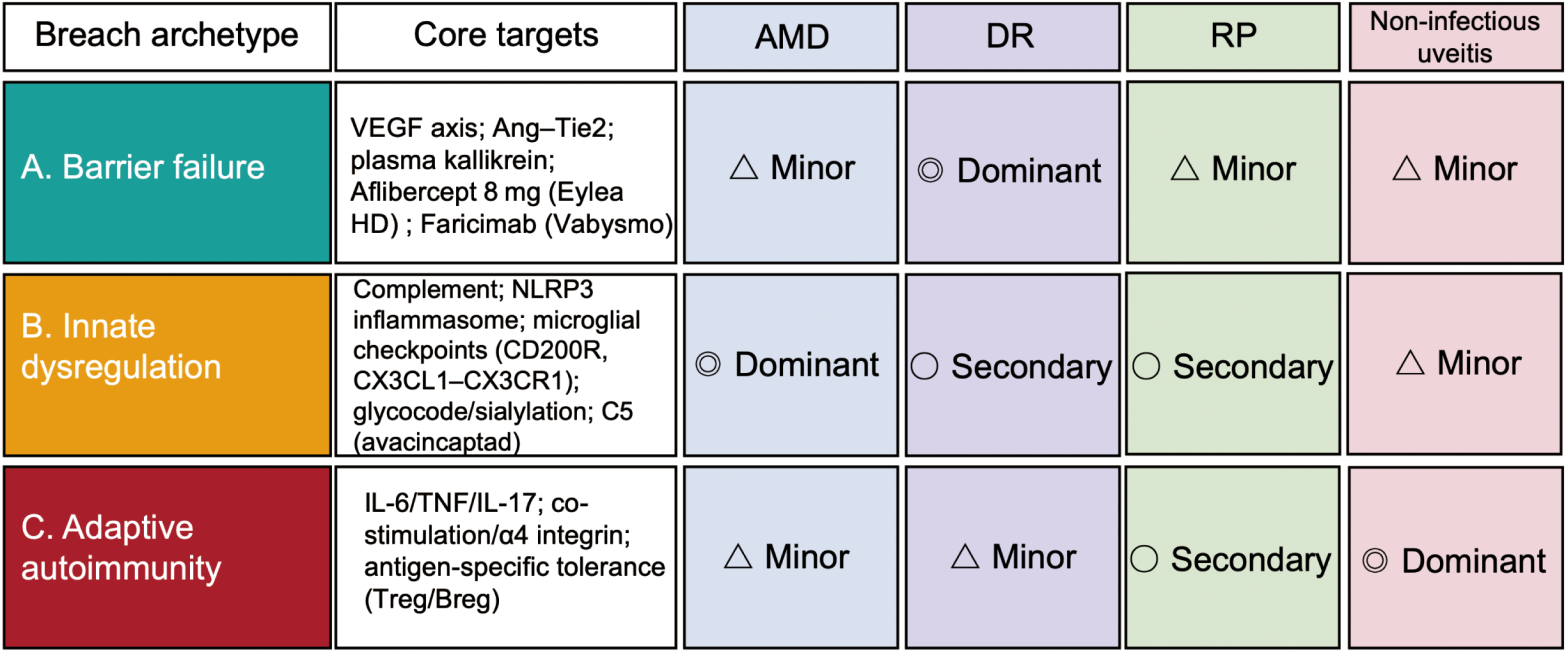
Therapeutic mapping to breach archetypes across four retinal diseases. Matrix view aligning mechanism-of-breach rows with disease columns (AMD, DR, RP, non-infectious uveitis), highlighting targets that **(A)** re-seal or stabilize barriers, **(B)** lower maladaptive innate set-points, and **(C)** constrain or re-educate adaptive immunity. Rows (mechanistic levers) **(A)** Barrier failure — agents that re-seal/stabilize the BRBs: VEGF axis, Ang–Tie2, plasma kallikrein.**(B)** Innate dysregulation — agents that lower myeloid/complement/inflammasome set-points: complement (C3/C5), NLRP3, microglial checkpoints (CD200R; CX3CL1–CX3CR1), and the “ glycocode”/sialylation axis. **(C)** Adaptive autoimmunity — agents that constrain pathogenic T cells and/or re-impose tolerance: IL-6/TNF/IL-17 blockade; co-stimulation/α4-integrin trafficking control; antigen-specific tolerance (Treg/Breg). Columns (diseases) Age-related macular degeneration (AMD), diabetic retinopathy (DR), retinitis pigmentosa (RP), and non-infectious uveitis (NI-uveitis). Evidence key Evidence status is harmonized with **Table 1** (interventional human / observational human / preclinical) and indicated in-figure. Symbols ◎ = dominant mechanism; ○ = secondary contribution; △ = minor/auxiliary contribution. color coding Teal = barrier; amber = innate; crimson = adaptive. Colors are harmonized with **Figure 1**; disease columns are subtly tinted for readability. Synthesis call-out Privilege restoration blueprint — re-seal/stabilized BRB, lower maladaptive innate set-point, re-educate adaptive immunity. This concept is supported by in-vivo evidence that the living eye can imprint regulatory/anergic programs on uveitogenic T cells. BRB, blood–retinal barrier; Ang–Tie2, angiopoietin–Tie2; NI-uveitis, non-infectious uveitis; Treg, regulatory T cell; Breg, regulatory B cell.

Retinal immunotherapy is shifting from one-size-fits-all suppression to mechanism-calibrated modulation that re-seals BRBs, quiets maladaptive innate programs, contains pathogenic T cells, and re-establishes tolerogenic circuits. Clinical validation already exists (e.g., anti-VEGF for leak/ischemia; C3 inhibition for GA; adalimumab for refractory uveitis) (80, 101). The next decade will likely refine microglial reprogramming, local gene-based immune control, and tolerance-oriented cell therapies, guided by biomarker-driven patient selection and the growing recognition that ocular immune privilege is dynamic—and can be therapeutically restored. A consolidated therapeutic map is provided in **Figure 3**, which integrates mechanisms, evidence type, and clinical readiness into a three-pillar blueprint for privilege restoration.

Evidence: Interventional human for anti-VEGF, steroid implants, C3 inhibition, anti-TNF, and IL-6R; Observational human for genetics/tissue, IL-6 correlation, pathway biomarkers, and biomarker-phenotype links; Preclinical for Wnt/β-catenin, adhesion axis, NLRP3, glycocode, inflammasome/IL-1 axis, co-stimulation/trafficking, Treg/Breg sufficiency, ocular imprinting, depletion, re-education, and AAV-mediated immunomodulation; Early clinical exploration for IL-6R and minocycline; Prospective validation needed for predictive biomarkers/genetics; Most emergent strategies remain preclinical or early clinical and warrant prospective trials.

## 10 Restoring ocular immune privilege: a translational roadmap

### 10.1 Rationale and organizing principles

Ocular immune privilege is not a fixed exemption from immunity but a therapeutically pliable state governed by barriers, innate immune set-points, and adaptive tolerance. Accordingly, restoration rests on three pillars: (i) re-sealing blood–retinal barriers (BRBs) to limit inflammatory flux; (ii) lowering maladaptive innate “noise” (complement, inflammasome, dysregulated microglia); and (iii) re-imposing regulatory programs on adaptive effectors, including antigen-experienced Tregs/Bregs and co-stimulation/trafficking control (21, 33–37, 69, 70, 80, 101).

### 10.2 Pillar II — quieting maladaptive innate programs (lower the inflammatory set-point)

Complement control. The success of C3 inhibition in slowing geographic atrophy validates complement as a tissue-damaging amplifier in AMD and a tractable therapeutic target.

Inflammasome/IL-1 axis. Caspase-1/IL-1β drives capillary degeneration in diabetes; NLRP3 inhibition (e.g., MCC950) reduces retinal leakage and inflammation *in vivo*, nominating inflammasome targeting for DR (and possibly late-stage RP) (75, 82).

Microglial tuning/checkpoints. Rather than indiscriminate depletion, checkpoint reinforcement can help restore homeostatic surveillance: AAV-delivered soluble CX3CL1 preserves cones and function in RP models (59); CD200R agonism restrains myeloid activation and angiogenic gene programs in ocular models (60). TGF-β signaling in retinal microglia is required to maintain quiescence; its loss precipitates degeneration and worsens neovascular sequelae, highlighting restorative TGF-β circuits (53).

Glycocode restoration. Age-related desialylation lifts brakes on complement and microglial phagocytosis; polysialic acid (polySia) reduces phagocyte activation and complement deposition *in vivo*, suggesting a surface “self-code” repair strategy for AMD (18).

### 10.3 Pillar III — re-educate adaptive immunity (from flare control to tolerance)

Cytokine interruption (e.g., TNF-α, IL-6 blockade) is effective in relapsing non-infectious uveitis, reducing flares and macular oedema (81, 101). Yet durable remission likely requires tolerance, not suppression alone. Classical ACAID/VCAID experiments show ocular antigens can elicit systemic deviation toward regulation (21, 33–38, 69, 70). In-vivo profiling now shows the living eye can imprint regulatory and anergic gene programs on uveitogenic T cells, indicating ocular immune privilege is active and, in principle, restorable—a mechanistic foundation for Treg/Breg-based therapies and antigen-specific tolerization. Adoptive transfer of antigen-experienced Tregs arrests retinal damage in spontaneous uveitis, while IL-35-induced Bregs suppress Th17/Th1 and confer resistance upon transfer (102, 103).

### 10.4 Disease-aligned playbooks

AMD (innate-dominant): Combine complement inhibition with microglial checkpoint reinforcement (CX3CL1/CD200R) and evaluate glycocode repair (polySia). In nAMD, anti-VEGF also re-seals the outer BRB, secondarily lowering inflammatory ingress (18, 49, 59, 60, 71, 72).

DR (barrier-first with innate amplification): prioritize barrier stabilization (anti-VEGF, steroids), then layer inflammasome/IL-1 and IL-6R modulation where cytokine profiles are elevated (19, 75, 80–82).

RP (degeneration-initiated, immune-amplified): Minocycline to damp microglia and CCR2^+^ monocyte influx; AAV-sCX3CL1 to deter maladaptive phagocytosis; reinforce TGF-β circuits for microglial restraint (27, 53, 59, 94).

Non-infectious uveitis (adaptive-dominant): TNF-α and IL-6R blockade for disease control; co-stimulation/adhesion strategies for trafficking-limited states; trajectory toward antigen-specific Treg/Breg interventions for durable tolerance (20, 81, 101).

### 10.5 Biomarkers and stratification

Genetics and fluids can guide allocation of mechanism-specific therapies: complement variants (CFH, C3, CFI) for complement targeting in AMD; aqueous/vitreous cytokines (IL-6/IL-8/CCL2) for inflammatory adjuncts in DR; T cell phenotypes and adhesion-molecule signatures to select uveitis biologics or trafficking blockers (76, 77). Emerging immuno-imaging and spatial single-cell metrics may supply on-treatment pharmacodynamic readouts.

### 10.6 Delivery and durability

Local, sustained delivery minimizes systemic exposure while resetting intraocular set-points: steroid implants for barrier repair; AAV vectors to express immune modulators (e.g., soluble CX3CL1) long-term; and biodegradable depots for cytokine antagonists (19, 59). Safety frameworks should include IOP monitoring, infection vigilance and steroid-sparing endpoints.

### 10.7 Trial design considerations

Outcome sets should triangulate structure(OCT for fluid/atrophy; wide-field ischemia), function(ERG, microperimetry)and immunobiology(aqueous cytokines, soluble complement, cell phenotypes). Enrichment by molecular driver(e.g., complement genotype, cytokine elevation)can increase power for mechanism-aligned therapies (76–80).

### 10.8 Outlook

Restoring ocular immune privilege is a pragmatic, mechanism-driven objective across retinal diseases. By re-sealing barriers, calming innate amplifiers and re-educating adaptive responses—ideally with biomarker-guided, locally delivered interventions—we can preserve vision while respecting the eye’s unique immune ecology. The convergence of gene delivery, cellular tolerance and checkpoint reinforcement, anchored by proof that the eye itself can imprint regulation, sets the stage for durable, disease-specific immune repair in the decade ahead.

Evidence: Interventional human anchors exist (anti-VEGF, steroid implants, complement inhibition, anti-TNF/IL-6R); Observational human for biomarker-phenotype links requiring prospective validation; Preclinical for Wnt/β-catenin, adhesion axis, inflammasome, microglial checkpoints, TGF-β circuits, glycocode repair, ACAID/VCAID, in vivo imprinting, Treg/Breg sufficiency, AAV, and depot platforms; Many tolerance-oriented and restorative strategies remain preclinical or early clinical; Adjuncts and methodological rationales warrant prospective, mechanism-stratified trials.

## 11 Conclusion

Retinal immune privilege is a dynamic, actively maintained—and potentially restorable—equilibrium that preserves vision by coupling surveillance to stringent inflammatory control. Across diseases, privilege fails through overlapping archetypes: innate para-inflammation at the RPE–Bruch’s interface in AMD, barrier-first failure with leukocyte ingress in DR, degeneration-led loss of regulation in RP, and T-cell–driven breach in autoimmune uveitis. Therapeutically, interventional human evidence now supports immunity as both driver and target: anti-VEGF re-seals leaky barriers, complement inhibition slows geographic atrophy, steroid implants suppress inflammatory oedema, and anti-TNF/IL-6R biologics improve refractory uveitis. The emerging paradigm is mechanism-aligned modulation—re-sealing BRBs, lowering maladaptive innate set-points, and containing or re-educating adaptive responses—rather than indiscriminate immunosuppression. Single-cell/spatial maps and in-vivo imprinting data sharpen this roadmap toward checkpoint reinforcement, inflammasome tempering, glycocode-based restraint, and tolerance-oriented strategies. Framed as a repairable regulatory state, ocular privilege invites biomarker-guided, locally delivered immunotherapy to control retinal inflammation and slow degeneration.
